# Rotavirus Surveillance at a WHO-Coordinated Invasive Bacterial Disease Surveillance Site in Bangladesh: A Feasibility Study to Integrate Two Surveillance Systems

**DOI:** 10.1371/journal.pone.0153582

**Published:** 2016-04-20

**Authors:** Arif Mohammad Tanmoy, ASM Nawshad Uddin Ahmed, Rajesh Arumugam, Belal Hossain, Mahfuza Marzan, Shampa Saha, Shams El Arifeen, Abdullah H. Baqui, Robert E. Black, Gagandeep Kang, Samir Kumar Saha

**Affiliations:** 1 Child Health Research Foundation, Department of Microbiology, Dhaka Shishu Hospital, Dhaka, Bangladesh; 2 Department of Pediatrics, Dhaka Shishu Hospital, Bangladesh Institute of Child Health, Dhaka, Bangladesh; 3 Department of Gastrointestinal Sciences, Christian Medical College, Vellore, India; 4 Department of Child and Adolescent Health, International Centre for Diarrhoeal Disease Research, Mohakhali, Dhaka, Bangladesh; 5 Department of International Health, Johns Hopkins Bloomberg School of Public Health, Baltimore, Maryland, United States of America; 6 Department of Microbiology, Dhaka Shishu Hospital, Bangladesh Institute of Child Health, Dhaka, Bangladesh; Second University of Naples, ITALY

## Abstract

The World Health Organization (WHO) currently coordinates rotavirus diarrhea and invasive bacterial disease (IBD) surveillance at 178 sentinel sites in 60 countries. However, only 78 sites participate in both surveillance systems using a common sentinel site. Here, we explored the feasibility of extending a WHO-IBD surveillance platform to generate data on the burden of rotaviral diarrhea and its epidemiological characteristics to prepare the countries to measure the impact of rotaviral vaccine. A six-month (July to December, 2012) surveillance, managed by IBD team, collected stool samples and clinical data from under-five children with acute watery diarrhea at an IBD sentinel site. Samples were tested for rotavirus antigen by ELISA and genotyped by PCR at the regional reference laboratory (RRL). Specimens were collected from 79% (n = 297) of eligible cases (n = 375); 100% of which were tested for rotavirus by ELISA and 54% (159/297) of them were positive. At RRL, all the cases were confirmed by PCR and genotyped (99%; 158/159). The typing results revealed the predominance of G12 (40%; 64/159) genotype, followed by G1 (31%; 50/159) and G9 (19%; 31/159). All in all, this exploratory surveillance collected the desired demographic and epidemiological data and achieved almost all the benchmark indicators of WHO, starting from enrollment number to quality assurance through a number of case detection, collection, and testing of specimens and genotyping of strains at RRL. The success of this WHO-IBD site in achieving these benchmark indicators of WHO can be used by WHO as a proof-of-concept for considering integration of rotavirus surveillance with WHO-IBD platforms, specifically in countries with well performing IBD site and no ongoing rotavirus surveillance.

## Introduction

Rotavirus (RV) is the leading etiological agent of severe diarrhea in young children [1]. Responsible for more than 120,000 of deaths and approximately 111 million episodes of gastroenteritis each year, rotavirus also necessitates more than 25 million clinic visits and 2 million hospitalizations each year [2]. Most of the deaths and severe diseases occur in low-income countries [3].

In the last two decades, improvements in oral rehydration solution use, access to healthcare and clean water supply with sanitation have reduced diarrheal incidence and mortality significantly in Bangladesh and many other countries [4, 5]. However, the incidence of RV infection has not declined substantially [6]. Such interventions do not have as much impact on rotavirus as these have on bacterial and parasitic agents [6], owing to the organism’s multiple modes of transmission [7] and ubiquity. Therefore, more targeted interventions, such as immunization, are needed to control RV gastroenteritis. Keeping that in mind, Bangladesh is also considering introduction of RV-vaccine in coming years.

Available RV-vaccines [8,9] have been demonstrated to be safe and highly efficacious (72–98%) in high- and middle-income countries [10–13]. However, in low-income countries of Asia and Africa, efficacy in clinical trials has been relatively low (42–63%) [14–16]. Although these vaccines show protection against a variety of circulating genotypes [17], the efficacy rate varies between industrialized and developing countries, due to many possible factors, including diverse RV-genotypes in the later [18–21]. In the past few decades, several strains have emerged in Asia and have spread globally [22], making it important to monitor strain ecology and understand the long-term interactions of strains with the available monovalent and multivalent vaccines [17].

Considering the high burden of this vaccine-preventable disease and the secular changes in strain circulation, the World Health Organization (WHO) began a global RV surveillance network of sentinel hospitals since 2008 to generate their own disease burden data and build the capacity to assess vaccine utility and impact. In addition to RV surveillance, the same team at WHO also coordinates the global invasive bacterial diseases (IBD) surveillance network at 178 sentinel sites in 60 countries [23].

These two surveillance network platforms, RV and IBD, have the same primary objectives: i) generating evidence for the introduction of vaccines into the national immunization program and ii) measuring vaccine impact in post-introduction period [23]. In spite of having identical objectives, only 23% (28/120) of surveillance sites in the Global Alliance for Vaccine and Immunization (GAVI) network provide data for both rotavirus and IBD. This ratio is even lower (12.5%; 1/8) in the SEARO region *[personal communication*, *Mary M*. *Agócs*, *WHO*. *4th Oct*, *2014]*. Integration of these two surveillance platforms together could make overall management easier and the surveillance less expensive through co-sharing study personnel and operating cost. In this study, we estimated burden and epidemiology of rotavirus using a high performing WHO-IBD sentinel site system to assess the feasibility of integrating these two surveillance platforms in the WHO network, specifically RV surveillance with ongoing IBD, using the performance indicators of WHO.

## Materials and Methods

### Study Site and Population

#### Site

Dhaka Shishu Hospital (DSH), a WHO-IBD surveillance site, is the largest pediatric hospital in Bangladesh and provides primary to tertiary care to urban and rural populations. Of the hospital’s 600 beds, 257 (43.6%) are reserved for families who are unable to pay. The hospital has a dedicated 18-bed ward for diarrheal patients. However, when the number of diarrheal patients is high, which occurs often, they are also admitted to general wards. WHO-IBD surveillance at this site enrolls 1,700 suspected IBD cases every year (unpublished data, Saha SK).

#### Population

The study was conducted among under-five children, admitted at the DSH, Bangladesh.

### Enrollment

For RV surveillance, all children aged between 0 and 59 months, with a primary diagnosis of diarrhea (3 or more loose/liquid stools within 24 hour period) by the treating pediatrician and admitted to the diarrhea ward during July to December 2012 were considered eligible for epidemiological data collection.

**Fig 1:**
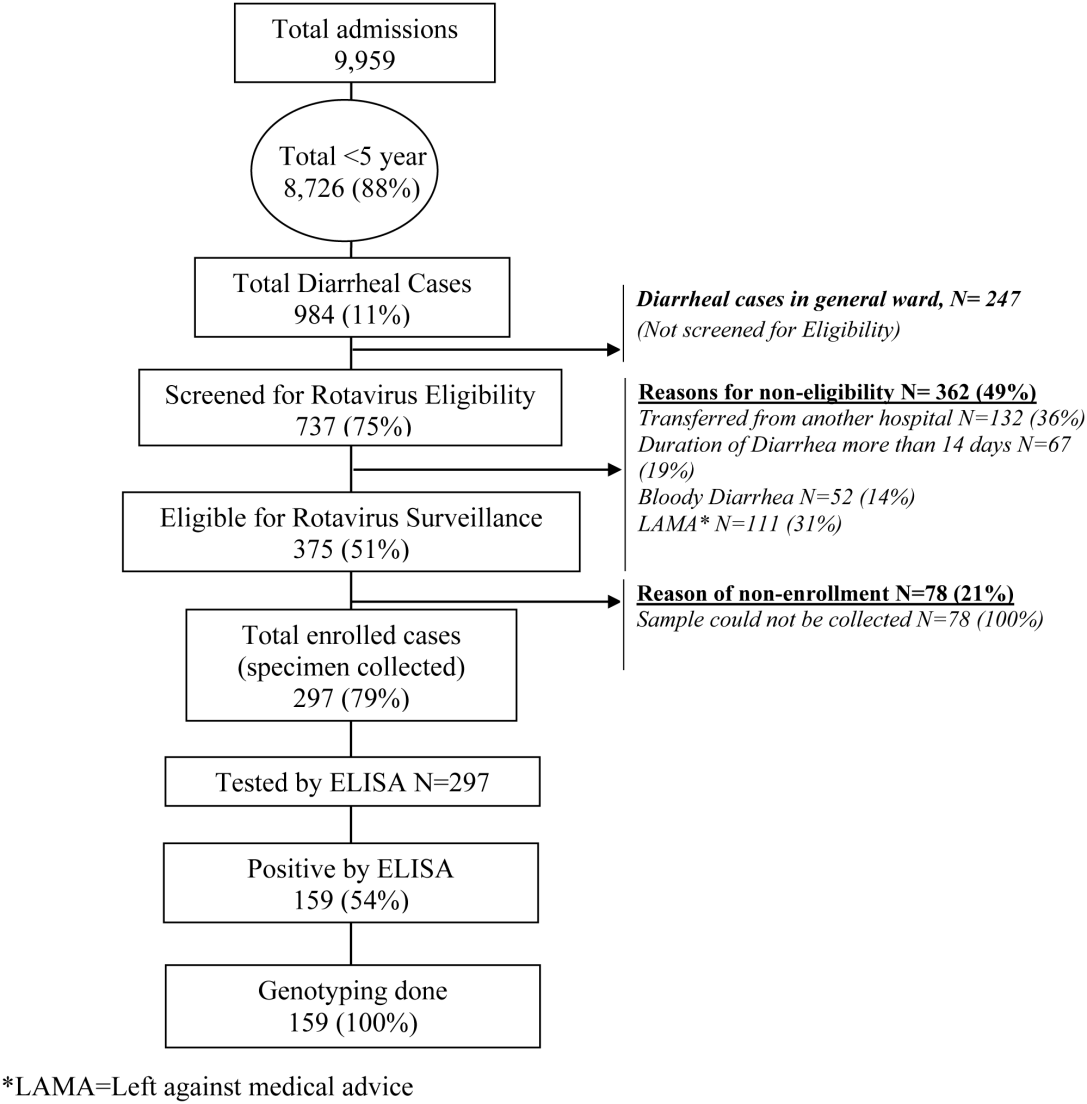
Rotavirus surveillance at Dhaka Shishu Hospital, Bangladesh during July-December, 2012.

Study physicians of IBD surveillance, with appropriate training on Rotavirus, screened the cases for acute watery diarrhea (duration <14 days) and enrolled them in the surveillance upon successful collection of stool specimens. Cases of dysentery and persistent diarrhea were excluded. Other inclusion and exclusion criteria are described in Fig 1. Study physicians obtained complete history of enrolled cases, performed physical examination, and recorded findings on the selected variables of IBD-case report forms. Enrolled patients were followed up by the study team until discharge and managed according to hospital protocol. The team also recorded the outcome of the patients at discharge.

#### Specimen Collection

Approximately 1–2 g of fecal specimens were collected from enrolled diarrheal cases on the day of hospital admission if possible, but no later than 48 hours from the time of admission. The suspension was vortexed and centrifuged, and the supernatant was preserved at 4–8°C for use within 30 days and at -70°C for long-term storage.

### Detection and genotyping

#### Detection of rotavirus

The supernatant (100μl) of the fecal suspension was used for detection of rotavirus-specific antigen using the ProSpecT Rotavirus ELISA test kit (Thermo Scientific, Waltham, MA, USA).

#### RNA Extraction, RT-PCR and Genotype-specific PCR

Viral RNA was extracted using the QIAxtractor (Qiagen, Hilden, Germany) platform. Extracted RNA was denatured at 97°C for 5 minutes and reverse transcription-PCR was performed using M-MLV reverse transcriptase (Invitrogen, Carlsbad, CA, USA) with random hexamers (Pharmacia Biotech, Uppsala, Norway) by following a single thermal cycle of 25°C for 5 minutes, 37°C for 60 minutes and 95°C for 5 minutes [24].

5μl of the cDNA was used for G-typing and P-typing PCR reactions of each specimen, following previously established procedures for eight G (1–4, 8–10, 12) types and six P (4, 6, 8–11) types rotavirus strains [25–26].

#### Microchip Electrophoresis Run

PCR products were run on a microchip electrophoresis system, MultiNA (MCE-202, Shimadzu Corporation, Japan) to visualize the bands and define rotavirus genotypes (G- and P-type) in respective specimens, based on band sizes. The whole genotyping procedure was performed at Welcome trust virology laboratory, Christian Medical College, Vellore, India (Regional Reference Laboratory for WHO RV surveillance).

### Feasibility of the IBD site to conduct Rota surveillance

Feasibility of a WHO-IBD site to conduct RV surveillance was weighed based on the performance indicators [27], used by WHO to monitor the performance of existing RV surveillance sites and decide their eligibility to continue. In addition, this study also collected the basic epidemiological data on RV cases to understand the capacity of the IBD site to gather comprehensive epidemiological and laboratory data.

### Data Analysis

The data were entered in EpiData version 3.1 and analyzed using STATA version 12.1.

### Ethics

The protocol and consent procedure for this short-duration RV surveillance was approved by the ethics review committee of the Bangladesh Institute of Child Health, DSH. As the sample collection protocol was not invasive, posed no threat to the patient’s health and no intervention was tried with the treatment, written consent was avoided as advised by the ethics committee. Instead, prior to enrollment, informed verbal consent from the patient’s parents/ caretakers was taken by the research assistant (RA) and documented on a consent log form with signature of both RA and a witness.

## Results

### Enrollment and Rotavirus detection

During the study period, 8,726 children (0–59 months) were admitted and 984 (11%) met the definition of diarrhea. Of those, 375 patients had acute watery diarrhea (AWD) and were eligible for inclusion in the study (Fig 1). Specimens were collected from 297 (79%) patients. All specimens were tested by rotavirus antigen ELISA; 159 (54%; 159/297) were positive (Fig 1).

#### Age and Sex Distribution

**Fig 2:**
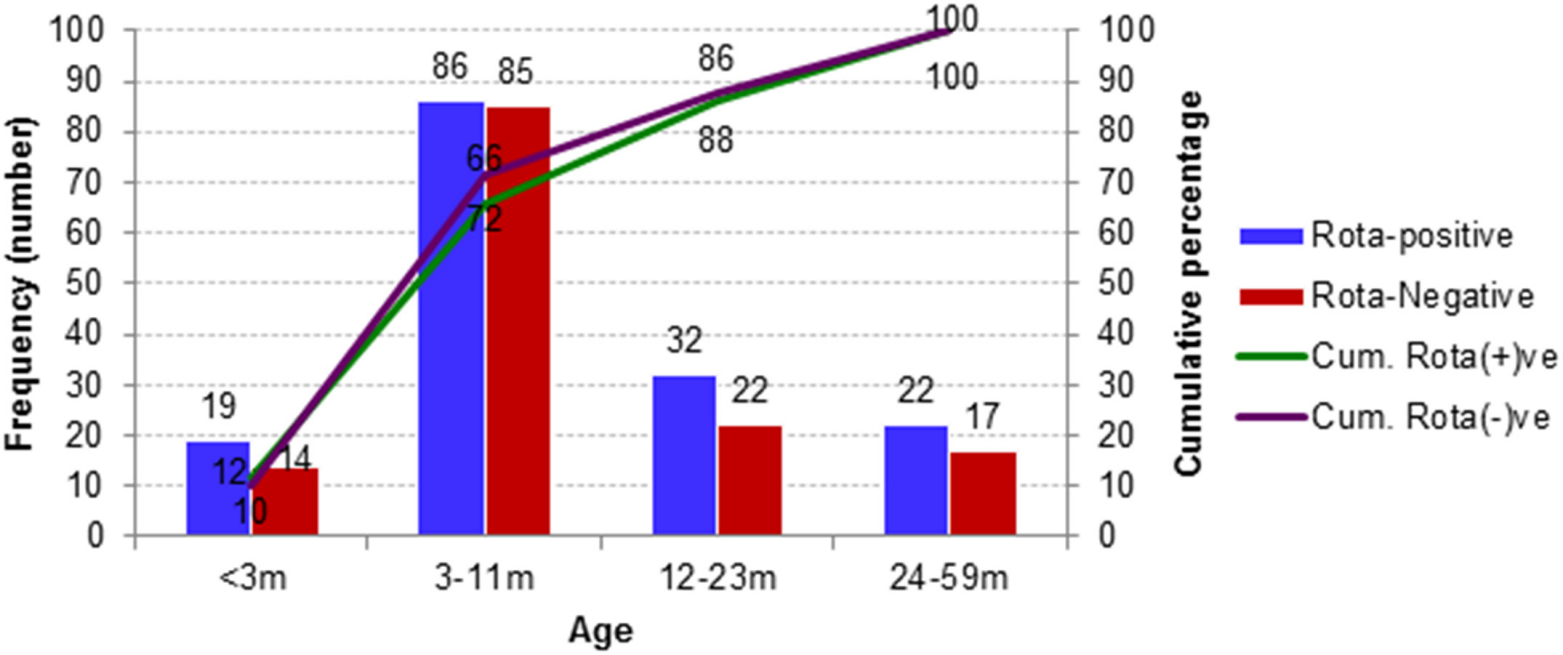
Age distribution of acute watery diarrhea cases with and without rotavirus.

Among the 159 rotavirus positive cases, 12% (19/159) was less than 3 months of age, while 66% (105/159) and 86% (137/159) were under 1 year and <2 years respectively (Fig 2). Males were 62% among both enrolled diarrheal (183/297) and rotavirus positive (99/159) cases.

#### Clinical Features

In addition to watery diarrhea, other common clinical manifestations in rotavirus positive cases were fever (16%; 25/159), vomiting (41%; 65/159) and dehydration (58%; 92/159). For most of the cases (89%; 141/159), diarrhea limited to 6 days or less (Avg. 3.61; SD ±1.99 days). Duration of fever ranged for 1 to 8 days (Avg. 2.24; SD ±1.5 days) and vomiting for 2 to 4 days (Avg. 1.8; SD ±0.9 days). No significant differences were found in clinical features between rotavirus-positive and rotavirus-negative diarrheal patients (Table 1; S1 Dataset). All patients were improved and none of them died.

**Table 1:** Clinical features of diarrheal cases with and without rotavirus.

Clinical features	Rota positive (N = 159)	Rota negative (N = 138)
*Diarrhea*	159 (100%)	138 (100%)
*Fever*	25 (16%)	21 (15%)
*Vomiting*	65 (41%)	57 (41%)
*Restless*, *irritable*	86 (54%)	84 (61%)
*Lethargic or unconscious*	6 (4%)	3 (2%)
*Some dehydration*	86 (54%)	84 (61%)
*Severe dehydration*	6 (4%)	3 (2%)

#### Genotypes

**Fig 3:**
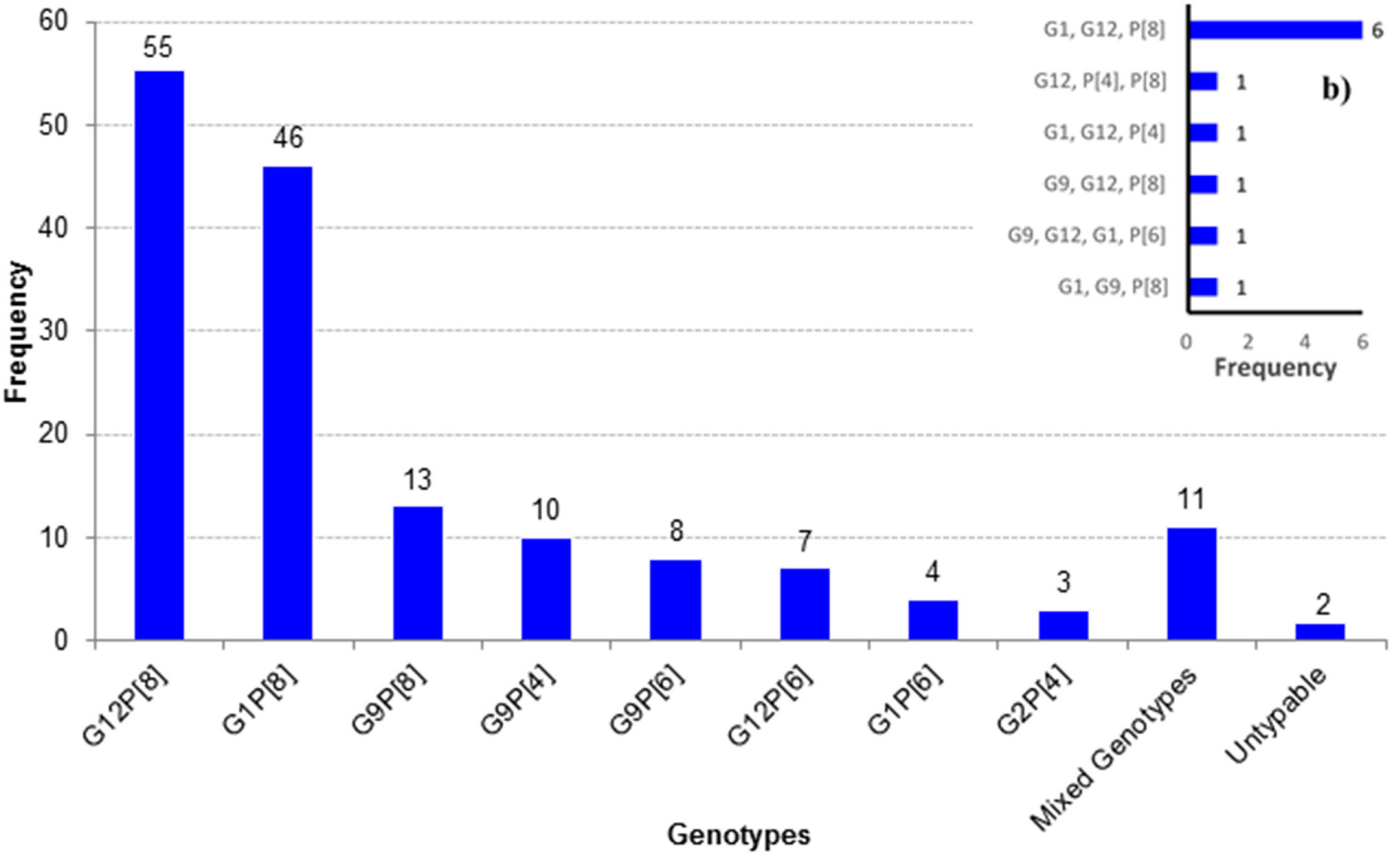
Circulating rotavirus genotypes; a) single genotype (N = 159), b) mixed genotypes (N = 11).

Genotyping revealed four G-types (G1, G2, G9 and G12) and three P- types (P[4], P[6] and P[8]). The most common G-type was G12 (40%; 64/159) followed by G1 (31%; 50/159), G9 (19%; 31/159) and G2 (2%; 3/159). The most prevalent P-type was P[8] with 77% (123/159), followed by P[6] (12%; 20/159) and P[4] (9%; 14/159). There were 55 cases of G12P[8] (35%; 55/159) with dominance (Fig 3). Other common G:P combinations were G1P[8] (29%; 46/159) and G9P[8] (8%; 13/159). Mixed genotypes were found in 7% (11/159) cases and two cases remained partly untypeable (Fig 3; S1 Dataset).

#### Monthly Variation of Rotavirus Cases

Over the study period, rotavirus infection peaked in November-December and again in July. Although diarrhea cases were lowest in October, rotavirus cases were lowest in the month of August.

### Feasibility to conduct rotavirus surveillance

A total of 297 specimens were collected during this 6 months pilot study, whereas WHO targets 100 specimens per year. Specimens were collected from 79% (n = 297) of eligible cases (n = 375); and 100% of them were within 2 days’ time. All (100%) specimens were tested by ELISA and rotavirus was detected in 54% (159/297) of enrolled cases. Again 100% of the positive cases were sent and confirmed in the RRL and 99% of them were genotyped by PCR (Table 2).

**Table 2:** WHO performance indicator criteria of rotavirus surveillance sites [27] and accomplishments of this pilot surveillance using WHO-IBD site.

WHO Criteria	WHO target	Our accomplishment in 6 months (and Comments)
Percent (%) of eligible children meeting the case definition that were enrolled with a case report form completed and specimen collected	80%	79% (297/375)
Percent (%) eligible enrolled acute diarrhea cases that were tested positive for rotavirus among cases who had stool specimens tested	20%	54% (159/297)
Percent (%) of cases with stool specimen collected within 2 days of admission	90%	100% (297/297)
Percent (%) of collected stool specimens that arrive at the laboratory for ELISA testing	95%	100% (297/297)
Percent (%) of received specimens that are tested in the site laboratory	90%	100% (297/297)
Percent (%) of rotavirus positive (ELISA confirmed) specimens sent to the RRL that are confirmed positive by the RRL	80%	100% (159/159)
Percent (%) samples genotyped in the RRL with results available at the site/country level within 6 months of sending specimens	90%	100% (159/159) (At real time)
Percent (%) of sites that report according to the agreed timeline for that site (at least quarterly)	80%	(Not applicable)
Number of enzyme immunoassay tests performed at the sentinel site	Minimum 100 specimens	297 (Six month surveillance)
The accuracy of rotavirus antigen detection testing by external quality assurance testing at the sentinel site	90%	100%
The score of the most recent WHO proficiency test as evaluated by on-site assessment	Min. 80%	Not applicable (Site is not assigned for Rotavirus surveillance)

## Discussion

Bangladesh and many other countries are considering the introduction of RV vaccine using funding from Gavi, the Vaccine Alliance. During this pre-vaccination phase, it is important to establish a platform for generating baseline burden of RV diarrhea and its epidemiological characteristics to prepare countries to measure the impact of the vaccine during post-vaccination years. Establishing a sustainable surveillance platform is an expensive and challenging endeavor. Leveraging the ongoing and well-functioning WHO-IBD sentinel site(s) for rotavirus surveillance would optimize available resources.

The six-month rotavirus surveillance using a WHO-IBD surveillance sentinel site platform successfully revealed a high burden of rotavirus in this tertiary-level hospital. The positivity rate (54%; 159/297) is much higher than those reported by other studies from Bangladesh (20% to 33%) [20–21, 28] and ongoing WHO surveillance sites in other countries (36%-42%) [23], but similar to reports from India (50%) [29], Myanmar (53%), Vietnam (59%) [30] and Libya (58%) [31]. This increased detection rate may be related to the time period of our study, which covered the months when rotavirus infection peaks, in the winter (December-February) and monsoon (July-August) seasons. During our surveillance, peaks of infection were found in November-December and July (data not shown) as seen in other studies [20–21, 28].

Seventy-four percent of rotaviral diarrhea cases were between three months to two years of age (Fig 2). This finding is consistent with previous studies from Bangladesh [20–21] and elsewhere [32], and provides evidence to support the addition of rotavirus vaccine to the present immunization schedule of Bangladesh at 6, 10, and 14 weeks.

Genotyping analysis of G and P types among detected rotavirus cases revealed eight different genotypic combinations, indicating a great diversity with the predominance of G12P[8] (Fig 3). In Bangladesh, the strain G12 was first detected at icddr,b sites (Dhaka & Matlab) during 2001–2006 and then 2006–2012 (Matlab site). During these two studies, the strain showed an increasing trend of prevalence, 5% to 15% [21,28]. Our study at DSH revealed G12 as the most predominant type (35%; 55/159), indicating the persistence of the strain for the last several years. The same strain was also found in 10% of cases in neighboring India, during 2005–2012 [33–34].

Among the P-types, P[8] is the most prevalent strain in this study, 77% (123/159). This is also true in India, though G12 strains were more commonly found in combination with P[6] than P[8] (188 vs 107), during 2005–12 [33–34]. In contrast, G12P[8] was the dominant genotype in the present study, compared to G12P[6] (35% vs 4%) (Fig 3).

As described above, our rotavirus surveillance using the platform and personnel of WHO-IBD study achieved all the performance indicators stipulated by WHO (Table 2), starting from enrollment of cases to genotyping of strains through sending the specimens to RRL and accuracy of rotavirus detection compared to RRL, etc within six months’ time (Table 2). All these comparative parameters indicate the feasibility of integrating rotavirus surveillance with an ongoing WHO-IBD site, specifically where RV surveillance is not in place.

This short-term study has the limitations of duration, but it clearly demonstrated the feasibility of generating data to fulfill pre- and post-vaccine introduction objectives of WHO rotavirus surveillance, including data for 1) describing disease epidemiology, including disease burden, 2) describing the circulating strains and their distribution, and 3) measuring vaccine impact after vaccine introduction [23], if pre-vaccine data can be collected for a full year or longer. Finally, success of this pilot study can be used by WHO and the countries as a demonstration in principle that a well performing WHO-IBD platforms, with minimal support, can generate comprehensive data on the pre-vaccine epidemiology of rotavirus, including disease burden and genotype distribution, and measure the post-vaccine impacts. However, WHO may consider conducting similar pilot studies at few other WHO-IBD sites in different countries to check the reproducibility and cost-effectiveness.

## Supporting Information

S1 Dataset Supporting rotavirus surveillance data file. Detail surveillance data including clinical features and genotyping results can be found in the “Rota Surveillance Data” sheet. A dictionary of the variables is also included in a separate sheet to explain the details.
(XLSX)
